# Supplementary material for: Near-visible integrated soliton microcombs with detectable repetition rates
Source: Nat Commun. 2025 May 22;16:4780. doi: 10.1038/s41467-025-60157-x (PMC12098722; doi:10.1038/s41467-025-60157-x)
Supplement: Supplementary file 1 — Supplementary Information [file 41467_2025_60157_MOESM1_ESM.pdf]

Supplementary Information:
Near-visible integrated soliton microcombs with detectable repetition rates

Peng Liu[1,*], Qing-Xin Ji[1,*], Jin-Yu Liu[1,*], Jinhao Ge[1,*], Mingxiao Li[2], Joel Guo[2], Warren Jin[2,3], Maodong Gao[1], Yan Yu[1], Avi Feshali[3], Mario Paniccia[3], John E. Bowers[2], Kerry J. Vahala[1,†]
[1]T. J. Watson Laboratory of Applied Physics, California Institute of Technology, Pasadena, CA 91125, USA
[2]ECE Department, University of California Santa Barbara, Santa Barbara, CA 93106, USA
[3]Anello Photonics, Santa Clara, CA, USA
[*]These authors contributed equally to this work.
[†]Corresponding authors: vahala@caltech.edu

This Supplementary Note is organized as follows. In Section I, a theory of dispersion design is presented. In Section II, eight supplementary data figures are provided to complement the main text. Figure S1 illustrates the experimental setups and parametric threshold measurements of the soliton microcombs in Fig. 2 of the main text. Figure S2 correlates the Kelly sidebands and dispersive waves in the 1064 nm soliton spectrum (middle panel of Fig. 2 of the main text) with corresponding dispersion measurements. Figure S3 summarizes phase noise measurements for the soliton microcombs in Fig. 2 of the main text. Figure S4 discusses anomalous dispersion designs for multiplexed solitons shown in Fig. 3 of the main text. Figure S5 presents the parametric threshold of the TM_0 mode 1064 nm soliton in Fig. 3 of the main text. Figure S6 summarizes phase noise measurements for the multiplexed soliton microcombs in Fig. 3 of the main text. Figure S7 presents a device design to achieve FSR-matched, polarization-multiplexed soliton microcombs at 1064 nm and 1560 nm bands. Figure S8 presents a device design to extend the color separation of multiplexed soliton microcombs to a full octave (780 nm and 1560 nm bands).

I. THEORY OF DISPERSION DESIGN

This section presents the theory for designing the anomalous dispersion window in Vernier-coupled ring resonators. Further details of the calculations can be found in [1]. Briefly, a transfer matrix, T, is used to propagate a two-component wave function through a round trip in the resonator:

$$T(m) \simeq e^{in_{\mathrm{wg}}(\omega_{m,\pm})\omega_{m,\pm}\overline{L}/c} \begin{pmatrix} e^{-i\omega_m \Delta L/c}\cos(g_{\mathrm{co}}L_{\mathrm{co}}) & i\sin(g_{\mathrm{co}}L_{\mathrm{co}}) \\ i\sin(g_{\mathrm{co}}L_{\mathrm{co}}) & e^{i\omega_m \Delta L/c}\cos(g_{\mathrm{co}}L_{\mathrm{co}}) \end{pmatrix} \tag{S1}$$

where $n_{\mathrm{wg}}(\omega_{m,\pm})$ is the effective index of the resonator waveguide, g_{co} is the coupling strength and L_{co} is the coupling length between the two rings, $\overline{L} = (L_A + L_B)/2$ is the average length and $\Delta L = (L_B - L_A)/2$ is the length difference.

The eigenfrequencies, $\omega_{m,\pm}$, of the two mode families (symmetric and antisymmetric branches) are determined by the secular equation:

$$\omega_{m,\pm} - \omega_m = \pm\frac{D_{1,m}}{2\pi}\arccos\left[\cos(g_{\mathrm{co}}L_{\mathrm{co}})\cos\left(2\pi\epsilon m\right)\right] \tag{S2}$$

where m is mode number, $\epsilon = \Delta L/\overline{L}$ is the length contrast of the rings, $\omega_m = 2\pi mc/(n_{\mathrm{wg}}\overline{L})$, c is the speed of light in vacuum, and $D_{1,m} = \partial\omega_m/\partial m$ is the first-order dispersion parameter.

The second-order dispersion parameter $D_{2,\mathrm{m},\pm}$ is obtained by differentiating $\omega_{m,\pm}$ twice with respect to m:

$$D_{2,m,\pm} - D_{2,\mathrm{o}} \approx \pm D_1 \frac{2\pi\epsilon^2\cos\left(g_{\mathrm{co}}L_{\mathrm{co}}\right)\sin^2\left(g_{\mathrm{co}}L_{\mathrm{co}}\right)\cos\left(2\pi\epsilon m\right)}{\left[1-\cos^2\left(g_{\mathrm{co}}L_{\mathrm{co}}\right)\cos^2\left(2\pi\epsilon m\right)\right]^{3/2}} \tag{S3}$$

where the coupling dispersion $\partial g_{\mathrm{co}}/\partial m$ is neglected, and $D_{2,\mathrm{o}} = \partial^2\omega_m/\partial m^2 < 0$ is the intrinsic second-order dispersion of the uncoupled resonator waveguide.

At the coupling center ($m = 0$), the maximal anomalous dispersion of the antisymmetric branch is given by:

$$D_2 = D_{2,m=0,+} = D_{2,\mathrm{o}} + \frac{2\pi\epsilon^2 D_1}{\tan(g_{\mathrm{co}}L_{\mathrm{co}})}. \tag{S4}$$

Equation S4 defines the maximal anomalous dispersion, a critical parameter for soliton generation.

II. SUPPLEMENTARY DATA FIGURES

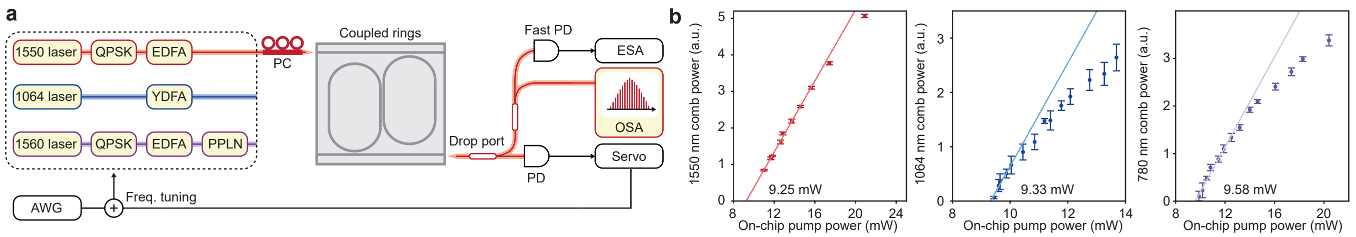

FIG. S1. **Soliton stabilization and parametric threshold measurements.** **a** Experimental setups for soliton measurements at 1550 nm, 1064 nm, and 780 nm. AWG arbitrary waveform generator, QPSK quadrature-phase-shift-keying, EDFA erbium-doped fiber amplifier, YDFA Ytterbium-doped fiber amplifier, PPLN periodically-poled lithium niobate, PC polarization controller, PD photo-detector, OSA optical spectrum analyzer, ESA electrical spectrum analyzer. The 1550 nm soliton is pumped by a sideband from an external cavity diode laser that is modulated by a quadrature-phase-shift-keying module. The sideband frequency is swept from blue-detuned to red-detuned regime to achieve the stable soliton states [2, 3]. The 1064 nm and 780 nm solitons use a similar approach, while the pump of the 780 nm soliton is frequency-doubled from an amplified 1560 nm external cavity diode laser. Calibrated on-chip pump powers are approximately 200 mW (1550 nm), 180 mW (1064 nm), and 190 mW (780 nm). **b** Measured parametric oscillation thresholds of 1550 nm, 1064 nm, and 780 nm coupled rings.

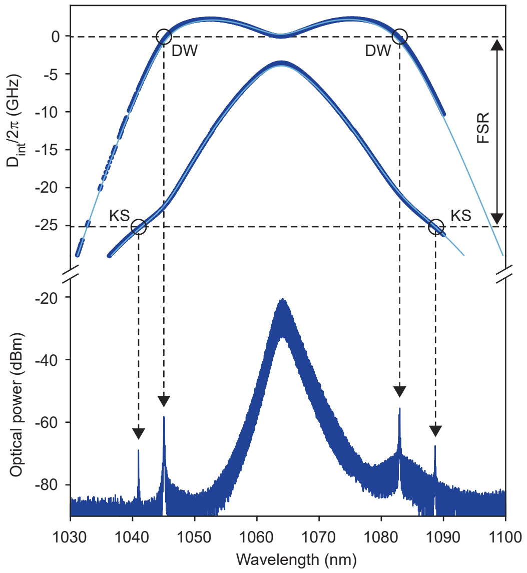

FIG. S2. **Dispersive waves and Kelly sidebands in the 1064 nm soliton microcomb.** The spectral position of two dispersive waves (DWs, intraband) and two Kelly sidebands (KSs, interband) agree well with dispersion measurements.

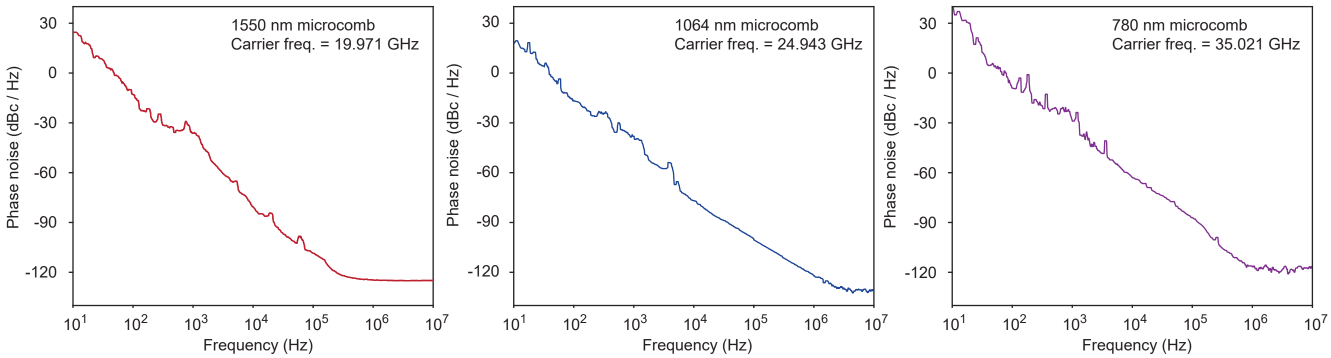

FIG. S3. **Phase noise measurements of microcomb repetition rate beatnotes.** The detected beatnotes of 1550 nm, 1064 nm, and 780 nm soliton microcombs are measured by a phase noise analyzer (Rodhe&Schwarz FSWP50) after appropriate microwave amplifiers.

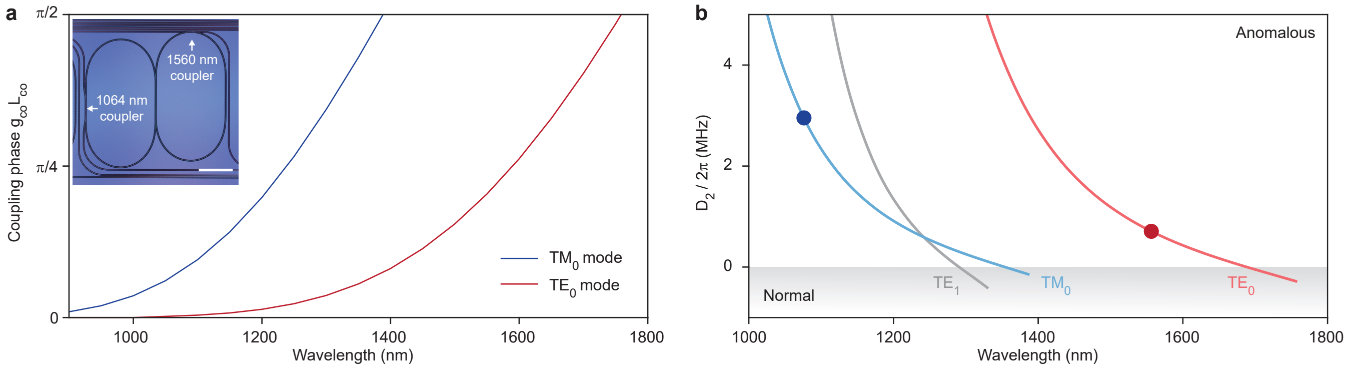

FIG. S4. **Coupling induced dispersion.** **a** The coupling phase $g_{\rm co}L_{\rm co}$ is larger at longer wavelength because of mode expansion. At a given wavelength, the coupling strength of $\rm TM_0$ is stronger than $\rm TE_0$, which underpins the approach for wavelength multiplexing of solitons in a single device. Inset: Photomicrograph of the wavelength-multiplexed coupled rings. Scale bar: 1 mm. **b** Simulated second-order dispersion coefficient D_2 of the coupled rings. Two high Q modes ($\rm TE_0$ and $\rm TM_0$) show anomalous dispersion for soliton generation from 1000 nm to 1700 nm bands. The operational points of 1064 nm and 1560 nm solitons are marked by dots.

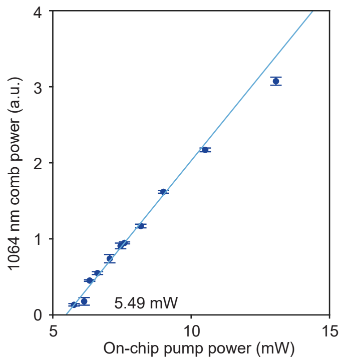

FIG. S5. **TM_0 mode parametric oscillation threshold power at 1064 nm.** The threshold power is measured in wavelength-multiplexed coupled rings with procedures detailed in Methods.

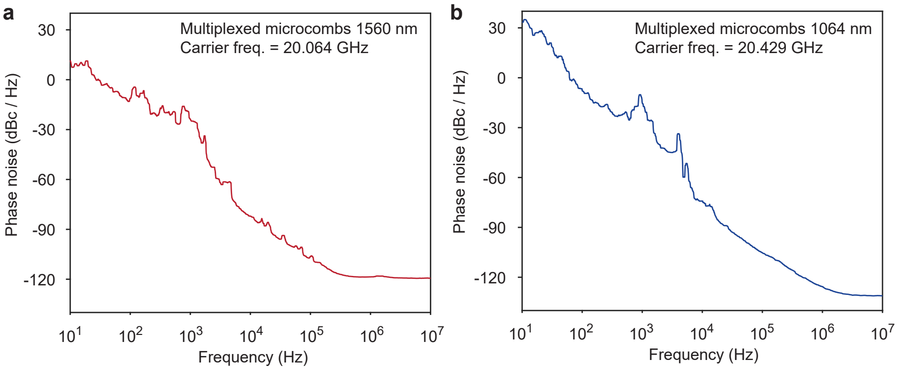

FIG. S6. **Phase noise measurements of multiplexed microcomb repetition rate beatnotes.** The detected beatnotes of 1560 nm and 1064 nm multiplexed soliton microcombs are measured by a phase noise analyzer (Rodhe&Schwarz FSWP50) after appropriate microwave amplifiers.

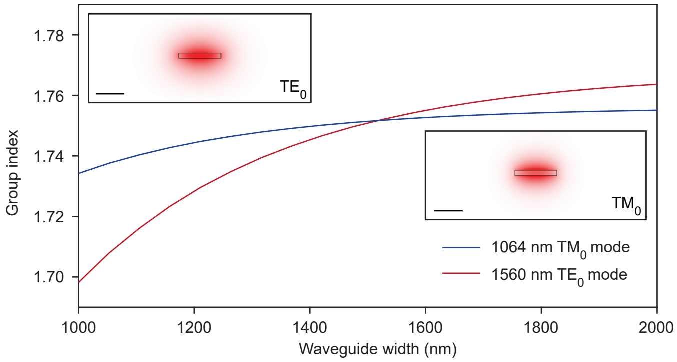

FIG. S7. **FSR matching of multiplexed soliton microcombs.** By tailoring waveguide width, the group index of 1064 nm TM_0 mode matches 1560 nm TE_0 mode in 185 nm thin-film silicon nitride waveguides (FSR matching point: 1518 nm), which indicates that FSR matching is feasible. Scale bar: 1 µm.

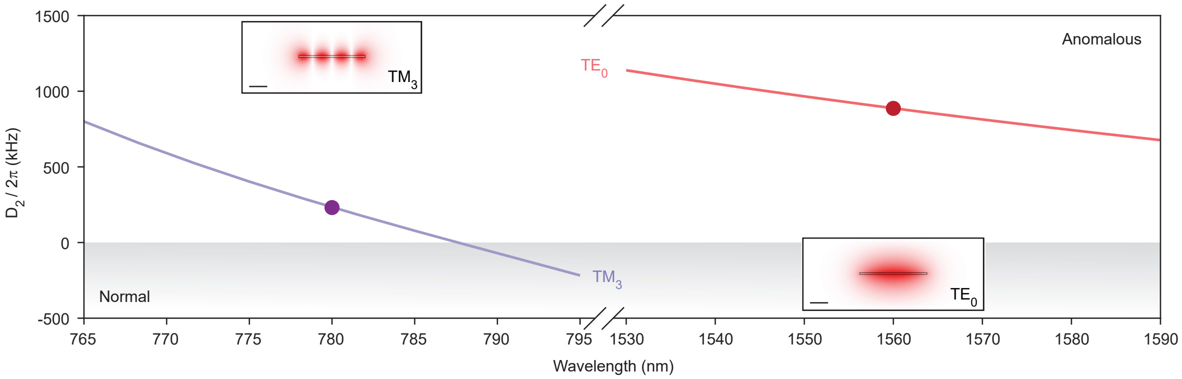

FIG. S8. **Extending multiplexed soliton microcombs to octave spacing.** By utilizing a higher order mode (TM_3), the anomalous dispersion window in thin-film silicon nitride waveguides is extended to the 780 nm band. Scale bar: 1 µm. This design is expected to support multiplexed soliton microcombs at 780 nm and 1560 nm simultaneously. Device dimensions used for simulation: silicon nitride thickness 100 nm, waveguide width 3700 nm, racetrack coupling gap 2200 nm, racetrack coupling length $L_{\mathrm{co}} = 1000$ µm, racetrack total length $\overline{L} = 9500$ µm, and length contrast $\epsilon = 1/400$.

[1] Yuan, Z. et al. Soliton pulse pairs at multiple colours in normal dispersion microresonators. Nature Photonics **17**, 977–983 (2023).
[2] Yi, X., Yang, Q.-F., Youl, K. & Vahala, K. Active capture and stabilization of temporal solitons in microresonators. Opt. Lett. **41**, 2037–2040 (2016).
[3] Stone, J. R. et al. Thermal and nonlinear dissipative-soliton dynamics in kerr-microresonator frequency combs. Phys. Rev. Lett. **121**, 063902 (2018).
